# ChromContact: A web tool for analyzing spatial contact of chromosomes from Hi-C data

**DOI:** 10.1186/s12864-015-2282-x

**Published:** 2015-12-15

**Authors:** Tetsuya Sato, Mikita Suyama

**Affiliations:** Medical Institute of Bioregulation, Kyushu University, Fukuoka, 812-8582 Japan; AMED-CREST, Japan Agency for Medical Research and Development, Fukuoka, 812-8582 Japan

**Keywords:** Hi-C, Long-range interaction, Enhancer

## Abstract

**Background:**

Hi-C analysis has revealed the three-dimensional architecture of chromosomes in the nucleus. Although Hi-C data contains valuable information on long-range interactions of chromosomes, the data is not yet widely utilized by molecular biologists because of the quantity of data.

**Results:**

We developed a web tool, ChromContact, to utilize the information obtained by Hi-C. The web tool is designed to be simple and easy to use. By specifying a locus of interest, ChromContact calculates contact profiles and generates links to the UCSC Genome Browser, enabling users to visually examine the contact information with various annotations.

**Conclusion:**

ChromContact provides wide-range of molecular biologists with a user-friendly means to access high-resolution Hi-C data. One of the possible applications of ChromContact is investigating novel long-range promoter-enhancer interactions. This facilitates the functional interpretation of statistically significant markers identified by GWAS or ChIP-seq peaks that are located far from any annotated genes. ChromContact is freely accessible at http://bioinfo.sls.kyushu-u.ac.jp/chromcontact/.

## Background

Hi-C can detect genome-wide three-dimensional (3D) chromatin interactions by cross-linking spatially proximal genomic fragments followed by high-throughput paired-end DNA sequencing [1]. Hi-C was successfully applied to unveil genome-wide long-range interactions [2] such as the relationship between distant enhancers and promoters. Recently reported Hi-C experiments identified a huge amount of spatial contact between genomic fragments, which contain known distant enhancers and their target genes, at 5–10-kb resolution [3]. More recent experiments achieved an even higher resolution [4].

Although the number of cell types with Hi-C data is limited, it is shown that megabase-sized chromatin domains are not significantly different between distinct cell types and even between species [2]; this indicates that to some extent the contact information obtained by Hi-C analysis can be transferred to other tissues or species to infer spatial contact in chromosomes from other samples. Moreover, the number of cell types with Hi-C data has recently expanded [4], and this will increase in the future.

Despite the fact that Hi-C data contains valuable information on 3D interactions within and between chromosomes, the data is not yet widely utilized by molecular biologists, mainly because of the quantity of data and complicated procedures used to calculate normalized contact matrices. To overcome this barrier, we developed a user-friendly web tool, ChromContact, for analysis of Hi-C data. Compared with currently available web-based analysis tools, such as 3DGD [5], the WashU Epigenome Browser [6], Juicebox [4], and Virtual 4C (http://promoter.bx.psu.edu/hi-c/virtual4c.php), ChromContact provides additional features including the following: (i) simple input specification; (ii) contact profile visualization in the UCSC Genome Browser via Track Hubs [7], which is capable of displaying several Hi-C data records for comparison with various annotations; (iii) analysis of multiple loci of interest at a single time; and (iv) output in text format for further downstream analyses.

## Implementation

We used high-resolution Hi-C data [4] deposited in the Gene Expression Omnibus (GEO) [8] with the accession number GSE63525. We downloaded contact matrices and normalization vector files registered under this accession to use in the ChromContact server. The current version of ChromContact covers six human cell types (GM12878, K562, IMR90, HUVEC, HMEC and NHEK) at three resolutions: 5 kb, 10 kb, and 25 kb. We used this data because it is the highest resolution Hi-C data available so far. We will update the data as new high resolution data is available. Contact matrices were normalized beforehand by applying normalization vector files. Because our main interest was to examine detailed structural interactions, such as enhancer–promoter interactions within a chromosome, we focused only on intrachromosomal interactions. Pre-calculated normalized contact matrices for a single sample occupy approximately 5.7 Gbyte (25-kb resolution) of server disk space. To extract contact information for a region of interest in a time- and memory-efficient manner, a random access function was implemented in our backend scripts.

Although Hi-C data is usually represented as a two-dimensional contact matrix, this makes it difficult to visually compare multiple Hi-C data with each other. Therefore, in ChromContact, we adopted 4C-like representation by specifying a user defined anchor coordinate to obtain a contact profile for the anchor, instead of directly using the two-dimensional contact matrix.

For the input, ChromContact uses a gene symbol, single nucleotide polymorphism (SNP) ID from a genome-wide association study (GWAS) Catalog [9], or a genomic coordinate. To analyze multiple loci, users may specify the input genomic coordinates in BED format, which is widely used in high-throughput genome analyses. In the case of gene symbol as an input, ChromContact automatically convert it to the coordinates of transcription start sites for the gene. The input coordinate is first converted to the corresponding anchor position, calculated based on the predefined resolution (5-, 10-, or 25-kb). A contact profile for the anchor (representing spatial proximity to the anchor for all chromosomal regions in a given resolution along the entire chromosome) is then generated from the normalized contact matrix.

In the option settings, the users may specify cell types and the resolutions to generate contact profiles. Although the highest resolution prepared in ChromContact is 5-kb for the contact profile, it often results in sparse data for the contact profile with many fluctuations, which makes it difficult to distinguish them from small but real peaks. For this reason, we adopted 10-kb resolution as a default parameter to obtain smoother contact profiles.

For the output, a link to the UCSC Genome Browser [10] containing the retrieved contact profile as the Track Hubs data [7] is generated to provide the user with a visual and interactive interface for detailed comparisons to up-to-date annotations stored in the UCSC Genome Browser. Since our main interest is to identify long-range enhancer–promoter interaction, ChromContact generates the Track Hubs data that contains not only the contact profile but also the profiles of H3K4me1 and DNaseI hypersensitivity sites by default. After opening the UCSC Genome Browser from the ChromContact output, the user can add other annotation tracks either by selecting the desired data stored in the UCSC Genome Browser or by uploading the user’s own data as a custom track. The user-friendly functionalities of the UCSC Genome Browser facilitate easy operation, for example zooming and scrolling, of the browser display.

For another output format, ChromContact also generates a list of regions with normalized contact counts in a text format, which facilitates further text-based downstream analyses; for example, sorting the results by contact counts using conventional spreadsheet applications.

## Results and discussion

**Fig. 1:**
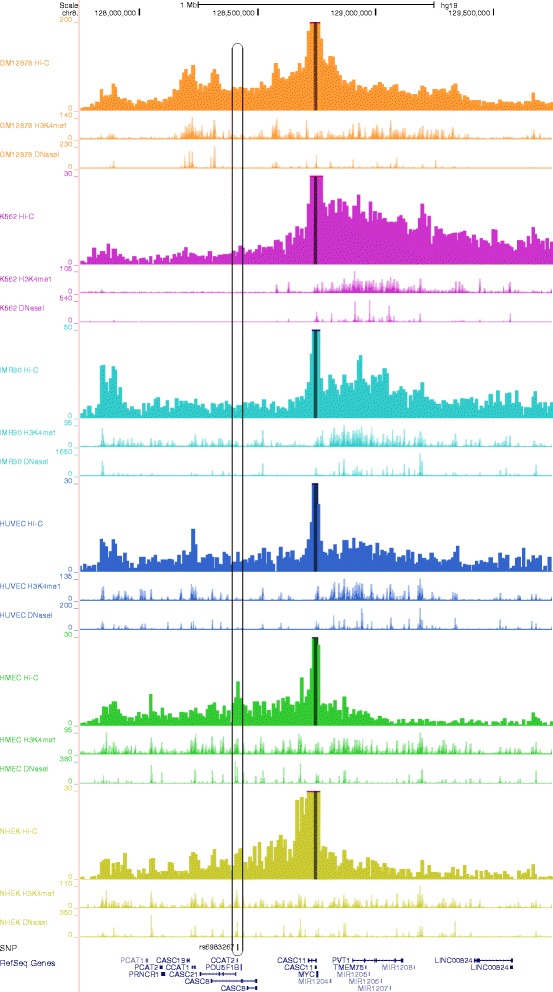
An example of a known long-range enhancer–promoter interaction in the human *MYC* locus. A 2-Mb genomic interval around the human *MYC* locus (genome assembly hg19) is shown in the UCSC Genome Browser. There are three tracks (from the top): (i) contact profile at 10-kb resolution obtained from Hi-C data; (ii) profile for H3K4me1; and (iii) profile for DNase I hypersensitivity sites, for each of the six cell types (GM12878, K562, IMR90, HUVEC, HMEC, and NHEK). Different colors are used for each of the six cell types. The anchor, which contains the transcription start site of *MYC*, is indicated by dark-colored highlights in the contact profiles. The colorectal cancer associated SNP, rs6983267, and gene annotations are shown at the bottom. The position of the SNP and the corresponding peaks in the profiles are indicated by a rounded rectangle

Here we demonstrate an example using the known long-range enhancer–promoter interaction in the human *MYC* locus (genome assembly hg19) (Fig. 1). SNP rs6983267, located 335-kb upstream of *MYC*, is strongly associated with colorectal cancer through spatial contact between SNP positioned in the *MYC* enhancer and promoter [11, 12]. By specifying the genomic coordinates of the *MYC* promoter as the anchor position and selecting all the six cell types for Hi-C data sets, ChromContact calculated the contact profiles for each of them. As shown in the UCSC Genome Browser output (Fig. 1), there is a prominent peak at the rs6983267 in the contact profile for HMEC cells (approximately chr8:128,413,000), indicating that the genomic fragment containing SNP rs6983267 can interact with the anchor (corresponding to the *MYC* promoter region) through chromosomal loop formation in HMEC cells. By comparing with ENCODE epigenomic data [13], H3K4me1, a histone mark for an enhancer, was observed in the same chromosomal position in HMEC cells, suggesting that the site may act as an enhancer. A DNase I hypersensitive site was also observed at this position. This indicates that the known distal enhancer–promoter interaction can be identified by incorporating currently available Hi-C and other epigenomic data. At the same genomic position, NHEK also has a peak in the profiles of Hi-C contact, H3K4me1, and DNase I hypersensitivity. The cell-type specificity of the Hi-C peak at this position well explain their association with colorectal cancer, i.e., both HMEC and NHEK belong to epithelial cells, from which most of colorectal cancer is developed. The same peak was not observed neither in hematopoietic cells (GM12878 and K562) and the other cell types (IMR90 and HUVEC). In the contact profiles, there are other peaks that correspond to H3K4me1 mark and DNase I hypersensitivity site in the matched cell types. These peaks may contain candidates for other *MYC* enhancers with cell-type specificity. As shown in this example, contact profiles are not always very similar between different cell types in rather high resolution such as 10-kb, but differ according to groups of cell types. This indicates that it is important to analyze Hi-C data from a cell type that is close to the one that the user is interested in, or to analyze Hi-C data from as many cell types as possible.

**Fig. 2:**
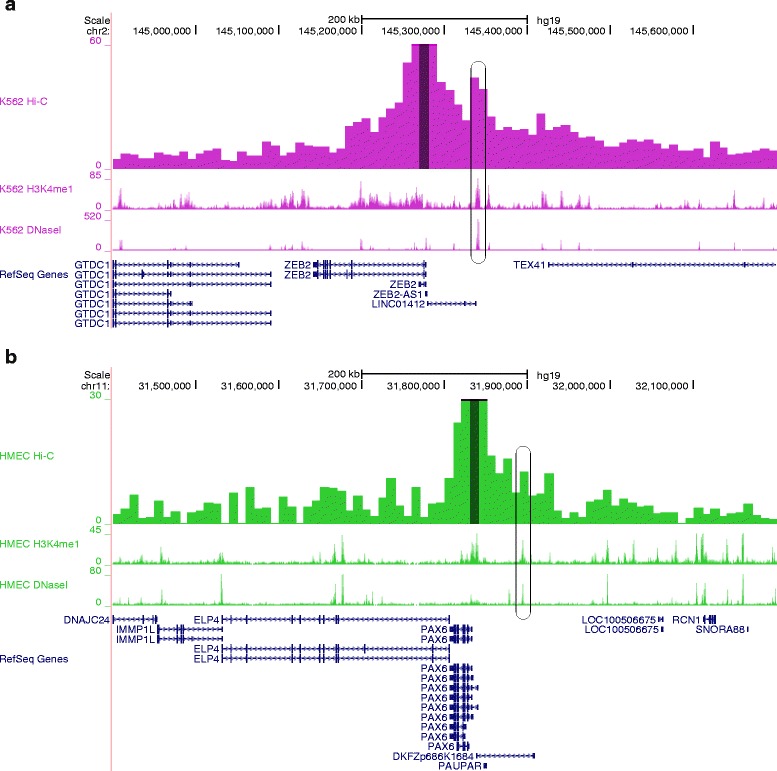
Two examples of long-range enhancer–promoter interactions in *ZEB2* and *PAX6* loci. Only the cell type with the most significant interaction is shown in each example. For each example, there are four tracks (from the top): (i) contact profile at 10-kb resolution obtained from Hi-C data; (ii) profile for H3K4me1; and (iii) profile for DNase I hypersensitivity sites; and (iv) gene annotations. The anchors, which contain the transcription start sites of the respective genes, are indicated by dark-colored highlights in the contact profiles. The positions of the enhancers are indicated by rounded rectangles. **a** A 800-kb genomic interval around the human *ZEB2* locus (genome assembly hg19). **b** A 800-kb genomic interval around the human *PAX6* locus (genome assembly hg19)

Next, we present two examples, for which long-range enhancer–promoter interactions had not been supported so far by 3C, 4C, or Hi-C experiments (Fig. 2). The first example is a long-range enhancer–promoter interaction found in *ZEB2* locus (Fig. 2a). It is reported that one of the enhancers for *ZEB2* conserved among human, mouse, and rat, and the enhancer is located about 65 kb upstream of the transcription start site of *ZEB2* in human [14]. In the contact profile, there is a peak that corresponds to H3K4me1 mark and DNase I hypersensitivity site, indicating that the enhancer region is spatially close to the promoter of *ZEB2* (Fig. 2a). The second example is a long-range enhancer–promoter interaction found in *PAX6* locus (Fig. 2b). There is a peak in the contact profile, which also corresponds to H3K4me1 mark and DNase I hypersensitivity site. For this region, enhancers for *PAX6* are identified in the corresponding region in mouse [15]. These data support the idea that the peak region contains enhancers for *PAX6* also in human, and that they are spatially proximal to the promoter of *PAX6* in HMEC cells.

## Conclusions

Increasing examples show that mutations not only in coding regions but also in regulatory regions are responsible for genetic diseases [16]. There are many cases in which statistically significant markers identified by GWAS or ChIP-seq peaks are located far from any annotated genes. Although there are already several known cases of long-range enhancer–promoter interactions, knowledge about these distal relationships is limited. This is mainly because identification of the relationships between distal regulatory elements and their target genes, which can be achieved via 3D architecture contact, has not been an easy task and requires laborious experiments. The recently devised Hi-C method, which reveals spatial interactions on a genome-wide scale, combined with rapid and continuous improvements of the method will dramatically change the means to analyze long-range interactions. ChromContact provides molecular biologists with a user-friendly means to access to high-resolution Hi-C data and facilitate analysis of long-range chromosomal interactions.

## Availability and requirements

Project name: ChromContact.

Project home page: http://bioinfo.sls.kyushu-u.ac.jp/chromcontact/.

Operating system(s): Platform independent (web-based).

Programming language: Perl, php.

Other requirements: None.

License: GPL-3.0.

Any restrictions to use by non-academics: No restrictions.

### Ethics statement

Ethics statement is not applicable to our study as this study only uses publicly available data.

## Abbreviations

3D: Three-dimensional
GWAS: Genome-wide association study
SNP: Single nucleotide polymorphism
